# Hospital Progress and Finance

**Published:** 1924-05

**Authors:** 


### HOSPITAL PROGRESS AND FINANCE.

### THE LISTER WARD.

Mr. James A. Morris, F.R.I.B.A., whose pamphlets on behalf of the retention of the Lister Ward at the Glasgow Royal Infirmary have done so much to explain the position, has not given up the struggle to preserve it. He suggests, in a recent letter, that the silence of the managers determined on demolition is due to a blind determination which hesitates to speak out because their principal argument, that the ward is in the way, cannot be reconciled with a proposal to build something else upon the site. They resent intervention from European and American critics because they fail to realise that they are trustees of a famous site in which the whole world is interested, and not merely its unrestricted owners. The workers are not, as has been asserted, unanimous in support of the policy of vandalism, and, at the moment of writing, the house-breakers at work on the block do not seem to have reached the famous ground floor. The reasons "compelling" destruction are still to seek, and the policy, unless prevented at the last moment, is to destroy, because the reasons against cannot be answered.

### HOSPITAL NEEDS OF THE WEMBLEY EXHIBITION.

To provide treatment for any visitors to the British Empire Exhibition at Wembley who may contract infectious disease during their visit, the Chiswick and Ealing Hospitals Committee have agreed to co-operate. It is planned to bring perhaps half a million children to the exhibition, and for their accommodation a private company has taken the old Filling Factory in Willesden Lane, Acton. The children are to be examined medically before their departure from home, so that an outbreak is very unlikely. We trust that this hope will be justified, for the annual plan of giving country holidays to town children has often caused epidemics in the villages to which they go, because, no doubt, medical inspection before their departure has been insufficient or neglected altogether. The enormous number of visitors to the Exhibition is bound to create risks to the public health, and it is satisfactory to know that some precautions have already been taken to minimise them.

### THE AMERICAN HOSPITAL AT HAMPSTEAD.

The American Hospital has been opened provisionally in a nursing home at Hampstead by the American Ambassador. The money in hand will enable the work to be carried on until experience teaches how many beds are needed by the American colony in London. These funds derive, at least in part, from the unspent balance still in the hands of the Care Committee for Soldiers and Sailors, which was formally handed over to the Provisional Committee of the hospital. A bronze slab accompanied the cheque to commemorate the work of the Care Committee during the war. All the American organisations in London have co-operated to make the new institution possible, and if the requirements prove a more ambitious hospital to be needed, the American colony will be asked to raise enough money to purchase or build such a hospital. The American Consul-General is the president of the Provisional Committee, and we shall await with interest the result of this scheme. More hospital beds are always needed, and London now falls into line with other capitals in having an American Hospital for the colony here.

### THE INSTABILITY OF REVENUE FROM TAXES.

In the April number of the *Empire Review*, Lord Hambleden, writing on hospital finance, states that the plan of devoting the proceeds of the entertainment tax to hospitals has been tried at Montreal, is not without its critics, and provides an income little more stable than that of voluntary contributions. This is an interesting experience, though mainly useful to defeat a proposal which, like the appropriation of any other tax, would probably be fatal to the voluntary system. It is not enough, however, to counter the case for nationalising hospitals on general grounds. Each proposal for supporting them out of the taxes needs to be answered separately; and the experience of Montreal, put forward by Lord Hambleden, is a serious objection to using for hospitals a tax which, from its incidence, seems to some an appropriate means of helping charity. Such attempts to make the impost fit the ideal are usually pre-doomed to failure, but it is satisfactory to learn that experience proves it. There are no financial short-cuts to the support of any necessary institution. The choice between voluntary effort and national support remains; and experience favours the former.

### A NEW HOSPITAL FOR LIVERPOOL.

We understand that an agreement has been reached to combine the Liverpool Hospital for Women, Shaw Street, and the Samaritan Hospital, Upper Parliament Street, and that the joint work will be carried on in a new building for which, with the aid of the Corporation, a site has been found at the junction of Bedford Street and Falkner Street. The Hospital for Women, which was established in 1841, has 59 beds, and the Samaritan Hospital 21. A joint committee is issuing an appeal for the new building, which will accommodate 120 beds and probably cost £100,000. Both hospitals have also deficits which it is hoped to meet by special subscriptions. It is not long since the Manchester hospitals for women amalgamated, and their example is encouraging a somewhat similar effort to place Liverpool in the same position.

### "INSURANCE" OF PRIVATE PATIENTS.

What sounds like a modification, amounting to a new departure, appears to have been adopted by the Royal Bucks Hospital at Aylesbury. With the idea of benefiting both patients and the hospital, it has been decided to admit patients of the class that pays income-tax to a special contribution scheme. The plan will be called a Private Patients

Scheme, and those who join it will be admitted as private patients to the hospital. The rates of payment are for a single person 10s. 6d. a year, for a married couple 21s., for a couple with children up to sixteen years £1 11s. 6d. Subscribers when ill will be admitted to the private wards at half the usual private ward charges. Thus we are a step nearer to that hospital provision for all classes which is the obvious goal to which experience, and the example of America, points. The only objection to a contribution scheme for millionaires or the "insurance" of those who pay super-tax is that these are relatively few and can afford existing nursing homes, which however as a rule are less efficient than the hospitals that do not admit the sick when unfortunate enough to be wealthy. But is there any reason why the hospitals should penalise the very rich when the special pavilions they might require could be built out of the fees appropriate to the super-tax payer ? There seems, by the way, to be no income limit for the Private Patients Scheme. Members of the ordinary scheme can be treated in the private wards at the full rate less 25 per cent. reduction.

### THE FAILURE OF LIVERPOOL.

The large deficit from which the Royal Southern Hospital, Liverpool, is suffering becomes more serious from the failure of the new method of raising funds by contributions. According to the hospital's president, Mr. Thomas Woodsend, the effort to start a contribution scheme in Liverpool, on the lines that have proved so successful in other parts of the country, has hitherto been unavailing. The system of mass contributions from employers and employed being the only known remedy for post-war expenditure, Mr. Woodsend hopes that the obstacles may be overcome. Liverpool and Manchester, we have often observed with regret, for some mysterious reason never seem to have been able to do what other towns have done for the support of voluntary hospitals. The present Lord Mayor of Liverpool, Mr. Arnold Rushton, is endeavouring with some others to devise a hospital fund. We, therefore, trust that he will insist that his support shall not be invoked for any momentary effort, but will stand for the right policy, which is to organise penny in the pound contributions from all businesses.

### ANOTHER HOSPITAL AMALGAMATION.

The Nottingham Castlegate and Samaritan Hospitals have severally agreed to a scheme of amalgamation. The name of the new institution will be the Castlegate and Samaritan Hospital for Women ; a site has been found, and the former has a sum of £11,000 for the new building. Mr. E. L. Manning, who has helped to devise the scheme, believes that it will produce an increased income by abolishing competition in the collection of funds. Economy and a better opportunity to specialise are also among the arguments. Unlike many schemes of the kind, this amalgamation seems to have been carried unanimously on both sides. Further details in regard to the new institution will be postponed until the necessary funds have been raised to complete it, though experience suggests that support may flag until the new building begins to rise, when there will be a desire to finish and pay for it.

### A GREAT CHANGE AT BRIGHTON.

Mr. Harry Preston has recently completed his year of office as president of the Royal Sussex County Hospital, and in this year the position of the hospital has been happily transformed. The total ordinary income was about £32,000, or £3,000 in excess of the ordinary expenditure. The average number of occupied beds was only 157, because during the first quarter 100 beds were closed, and a grant of some £4,000 from the Voluntary Hospitals Commission has been given on condition that all the beds are kept open during 1924. The support gained is said to be unprecedented, and for this turn of the tide Mr. Ball Dodson, the retiring chairman of the Board, and Mr. Preston himself deserve every congratulation. The loss of both in their responsible offices will be great, but they have accomplished a fine task, especially during the past twelve months.

### ALMONERS, CHILDREN AND THE LONDON COUNTY COUNCIL.

The value of the special schools at the disposal of the London County Council for children whom some defect keeps away from the ordinary elementary schools, is the subject of a pamphlet by Sir William Hamer, Medical Officer of Health and School Medical Officer to the Council. The pamphlet is being distributed to hospitals for the information of the medical staffs and almoners in the hope that they may overcome the prejudice that certain parents feel against sending their children to special schools. Such schools exist for blind, deaf, mentally defective, myopic and stammering children ; there are open-air schools also, and the Council can enter epileptic children at special residential schools outside the metropolis. As it is, many children who would benefit by the facilities of these special schools remain at an elementary school or stay away altogether.

### A VETERAN ADMINISTRATOR.

Unusual interest was given to the annual meeting of the London Homœopathic Hospital by certain announcements and a particular presentation that were made. The Prince of Wales has been asked to become patron and the Duke of York president of the hospital, and Lord Donoughmore, who is the treasurer, believes that these requests are being considered favourably. The meeting closed with the presentation to the secretary, Major Edward Attwood, of a keyless gold watch and chain, subscribed by the members of the board. The watch bears the inscription : "Major E. A. Attwood, from the board of management, to commemorate 40 years' service with the London Homœopathic Hospital, 1884-1924." This is a fine record, and its acknowledgment coincides with a substantial advance toward the additional annual subscriptions that were asked for a year ago. Major Attwood has been a tireless worker on the hospital's behalf, and all who have come in contact with him will rejoice in the latest proof of confidence and gratitude that he has received.

